# Sunlight-Driven AO7 Degradation with Perovskites (La,Ba)(Fe,Ti)O_3_ as Heterogeneous Photocatalysts

**DOI:** 10.3390/nano11113142

**Published:** 2021-11-21

**Authors:** Ana Sofia Rodrigues, Lurdes Ciríaco, Maria José Pacheco, Annabel Fernandes, Sandra Mogo, Ana Lopes

**Affiliations:** 1 Fiber Materials and Environmental Technologies (FibEnTech-UBI), Universidade da Beira Interior, R. Marquês de D’Ávila e Bolama, 6201-001 Covilhã, Portugal; ana.sf.rodrigues@ubi.pt (A.S.R.); lciriaco@ubi.pt (L.C.); mjap@ubi.pt (M.J.P.); analopes@ubi.pt (A.L.); 2 Department of Physics, Universidade da Beira Interior, 6201-001 Covilhã, Portugal; smogo@ubi.pt; 3 Atmospheric Optics Group, University of Valladolid, C/Plaza de Santa Cruz, 8, 47002 Valladolid, Spain

**Keywords:** perovskites (La,Ba)(Fe,Ti)O_3_, photocatalysis, sunlight, AO7, ceramic method, complex polymerization

## Abstract

Perovskites of the (La,Ba)(Fe,Ti)O_3_ family were prepared, characterized, and utilized as heterogeneous photocatalysts, activated by natural sunlight, for environmental remediation of Acid Orange 7 (AO7) aqueous solutions. Catalysts were prepared by the ceramic (CM) and the complex polymerization (CP) methods and characterized by XRD, SEM, EDS, and band gap energy. It was found that catalytic properties depend on the synthesis method and annealing conditions. In the photocatalytic assays with sunlight, different AO7 initial concentrations and perovskite amounts were tested. During photocatalytic assays, AO7 and degradation products concentrations were followed by HPLC. Only photocatalysis with BaFeO_3_-CM and BaTiO_3_-CP presented AO7 removals higher than that observed for photolysis. However, photolysis leads to the formation of almost exclusively amino-naphthol and sulfanilic acid, whereas some of the perovskites utilized form less-toxic compounds as degradation products, such as carboxylic acids (CA). Partial substitution of Ba by La in BaTiO_3_-CM does not produce any change in the photocatalytic properties, but the replacement of Ti by Fe in the La_0.1_Ba_0.9_TiO_3_ leads to reduced AO7 removal rate, but with the formation of CAs. The best AO7 removal (92%) was obtained with BaFeO_3_-CM (750 mg L^−1^), after 4 h of photocatalytic degradation with solar radiation.

## 1. Introduction

Photocatalytic oxidation technology has high potential for the degradation of the wastewater’s organic load due to its high efficacy and energy-saving advantages, wide application range, and no secondary pollution [1,2,3]. If powered by solar energy, a type of clean energy, with an appropriate catalyst, sunlight-driven photocatalysis can be an attractive approach for environmental remediation [4].

Among heterogeneous photocatalysts, perovskite oxides, with high structural stability even under aggressive conditions, a flexible chemical composition, and elemental abundance, are regarded as promising catalysts for many different reactions [5,6,7,8]. They are a class of compounds presenting the general formula ABO_3_, where A is an alkali or alkaline earth metal or a member of the lanthanides’ family and B a transition metal [5,9]. The network oxygen in the perovskite’s structure exhibit high mobility within perovskites, allowing vacancies and the stabilization of uncommon metal oxidation states [8,10]. Thus, these perovskite oxides offer a rich opportunity for designing novel structures with unique properties by introducing different metal ions into the structural framework [9,11,12,13]. The partial substitution of the cation sites (A or B) with additional cations (A′ or B′) results in A_x_A′_1-x_B_y_B′_1-y_O_3-δ_ compounds [14]. Although in the general perovskite formula δ = 0, many perovskite oxides are oxygen-deficient due to the A to B molar ratio, respective sizes, electronic configurations, and coordination numbers, making δ > 0 [15].

To be utilized as photocatalysts, perovskite-type metal oxides must present semiconductor properties, i.e., a band gap energy (Eg) between 1.4 and 3.8 eV. Most of the perovskites with titanium in the B position show exceptional photocatalytic properties under UV radiation since they present Eg values higher than 3.0 eV. Other perovskites, such as FeTiO_3_, with Eg of 2.8 eV, and LaFeO_3_, with 2.1 eV, may absorb visible light [9,16,17]. In fact, for good environmental practices, it is important to develop photocatalysts that work under visible light to use the maximum potential of solar energy [18]. Perovskites are optimum candidates for this development since their optical properties may be changed by doping, thus inducing visible light absorption [9]. This would allow their utilization with sunlight, a green, safe, and sustainable energy, which is composed by ultraviolet (8%), visible (40%), and infrared (52%) radiations [19].

Some perovskite oxides were already utilized in the photocatalytic degradation of several dyes by utilizing natural [20,21,22] or simulated sunlight [23] or visible light [24,25,26,27]. In fact, perovskites have the most intriguing physicochemical features that allow researchers a wide range of tunability to obtain high photocatalytic efficiency, stability, and low-rate electron-hole recombination [18,28].

The photocatalytic activity of some perovskite materials may also be enhanced by the presence of heterostructures, as in the Acid Orange 7 (AO7) photodegradation study by visible light with Sr_0.95_Bi_0.05_TiO_3_ and Bi_4_Ti_3_O_12_ phases [29] or by heterojunction photocatalysts, as in the Rhodamine B degradation using as photocatalyst LaFeO_3_/BiOBr activated by simulated sunlight as a radiation source [30].

Therefore, the aim of this work was to prepare perovskites of the La_x_Ba_(1-x)_Fe_y_Ti_(1-y)_O_3_ family, synthesized by ceramic or polymerization complex methods, at different annealing conditions and compare their photocatalytic activity in suspension for the degradation of the model dye, the azo dye AO7, with natural sunlight. Since AO7 degradation may originate sulfanilic acid (SA) and 1-amino-2-naphthol (AN) as by-products, which are very harmful to the environment, particularly AN, an unstable aromatic amine [31], these by-products were also monitored during AO7 photocatalytic degradation, as well as some carboxylic acids, to assess not only AO7 photodegradation’s immediate metabolites but also to predict SA and AN photocatalytic degradation.

## 2. Materials and Methods

Perovskite oxides of the family La_x_Ba_(1-x)_Fe_y_Ti_(1-y)_O_3_, namely BaTiO_3_, BaFeO_3_, La_0.1_Ba_0.9_Fe_0.6_Ti_0.4_O_3_, La_0.1_Ba_0.9_Fe_0.4_Ti_0.6_O_3_, La_0.1_Ba_0.9_TiO_3_, and LaFeO_3_, were prepared utilizing the ceramic method (CM) [32]. FeTiO_3_ was also prepared since it contains the two metal ions of position B of the perovskite family under evaluation. For the synthesis using the ceramic method, stoichiometric amounts of commercial La_2_O_3_ (+99.9%, Acros Organics, VWR International, Amadora, Portugal), BaCO_3_ (+99.0%, Fluka, VWR International, Amadora, Portugal), Fe_2_O_3_ (+99.0%, Merk, Lisbon, Portugal) and TiO_2_ (+99.8%, Sigma Aldrich, Lisbon, Portugal) were weighed, milled for 30 min, pre-calcined at 900 °C in a tubular furnace for 6 h, and calcined at 1130 °C for 4 h. To study the influence of the calcination time on BaTiO_3_ properties, the calcined mixture was milled for 15 min and subjected to a 24 h extra calcination period at 1130 °C.

The perovskite BaTiO_3_ was also synthesized by the complex polymerization method (CP) [33,34], using stoichiometric amounts of commercial BaCO_3_ (+99%, Fluka, VWR International, Amadora, Portugal) and C_12_H_28_O_4_Ti (+98%, Acros Org., VWR International, Amadora, Portugal). Initially, C_12_H_28_O_4_Ti was dissolved in ethylene glycol (+99,5%, Carlo Erba, LaborSpirit, Loures, Portugal), and then citric acid (+99%, Sigma Aldrich, Lisbon, Portugal) was added, being ethylene glycol/citric acid volumetric ratio 1:4. After that, BaCO_3_ was added, and the resulting mixture was submitted to successive heating: at 50 °C, then at 90 °C, and after that at 150 °C, for 20 min each; at 400 °C, for 2 h, in a tubular furnace, with a heating rate of 5 °C min^−1^, for the formation of a black precursor powder, and after it was calcined twice at 900 °C, for 3 h (5 °C min^−1^), being ground between the two thermal treatments.

All the synthetized powders were characterized by X-ray diffraction (XRD), dispersive energy spectroscopy (EDS) and scanning electron microscopy (SEM). Perovskite X-ray diffraction analysis was performed using a Rigaku diffractometer, model DMAX III/C, with automatic data acquisition (MDI, Materials Data), equipped with a monochromatized Cu kα radiation (λ = 0.15406 nm), operating at 40 mA and 30 kV. The crystallite’s dimension was calculated by means of the Scherrer equation. EDS and SEM characterizations were done in a Hitachi S-3400 N/Bruker system (Monocomp Instrumentación S.A., Madrid, Spain), operating at 20 keV. Diffuse reflectance spectra of the perovskite films were also obtained, in a UV−vis spectrometer Shimadzu UV-2600 PC (Izasa Scientific, Carnaxide, Portugal), equipped with an integrating sphere ISR 2600 plus, over the spectral range 200−900 nm. Kubelka−Munk function was used to analyze the diffuse reflectance spectra. BaSO_4_ was utilized as a reflectance standard.

Perovskite powders’ efficiency as heterogenous photocatalysts was tested in simultaneous degradation assays under natural sunlight. The photocatalytic assays were performed in batch mode with orbital stirring, using 50 mL of the AO7 solution, for 4 h. Different AO7 initial concentrations (5, 10, and 20 mg L^−1^) were tested. The influence of perovskite powders amount was also investigated (250, 500, and 750 mg L^−1^). For some chosen perovskites, 200 mL assays were also run.

AO7 degradation tests were monitored by UV–vis absorption spectrophotometry (Shimadzu UV-vis 1800 spectrophotometer, Izasa Scientific, Carnaxide, Portugal) at wavelengths ranging from 200 to 800 nm. The identification of AO7 and some metabolites, such as sulfanilic acid and 1-amino-2-naphthol, was performed with a Shimadzu 20 A Prominence system (Izasa Scientific, Carnaxide, Portugal), equipped with an SPD-M20A diode array detector. Chromatographic separations were carried out using a reverse phase Purospher STAR RP-18 endcapped column (250 mm × 4 mm (i.d.), 5 μm particle size) (Merck Millipore, Algés, Portugal), and a mixture of phosphate buffer (33 mM) at pH 7 (solvent A) and acetonitrile (solvent B) as the mobile phase, with a flow rate of 0.7 mL min^−1^, and using a gradient elution: 20% A: 80% B at the initial 9 min; 40% A:60% B at 11 min, and this composition was maintained until the end of the run (35 min). The injection volume was 20 μL, and the column temperature was 35 °C. The wavelengths (in nm) and the retention times (in min) utilized in these determinations were: AO7—484, 21.1; SA—249, 2.9; AN—252, 18.4.

In the treated solutions, some carboxylic acids were also identified by ion-exclusion chromatography, as described elsewhere [35], being the retention times for the different acids, in minutes, as follows: oxalic acid—6.4; maleic acid—7.8; oxamic acid—8.9; formic acid—13.4; acetic acid—14.6.

Sunlight irradiance was measured with a Newport 835 Optical Power Meter (M.T. Brandão, Lda, Porto, Portugal).

## 3. Results and Discussion

### 3.1. Catalysts Characterization

**Figure 1:**
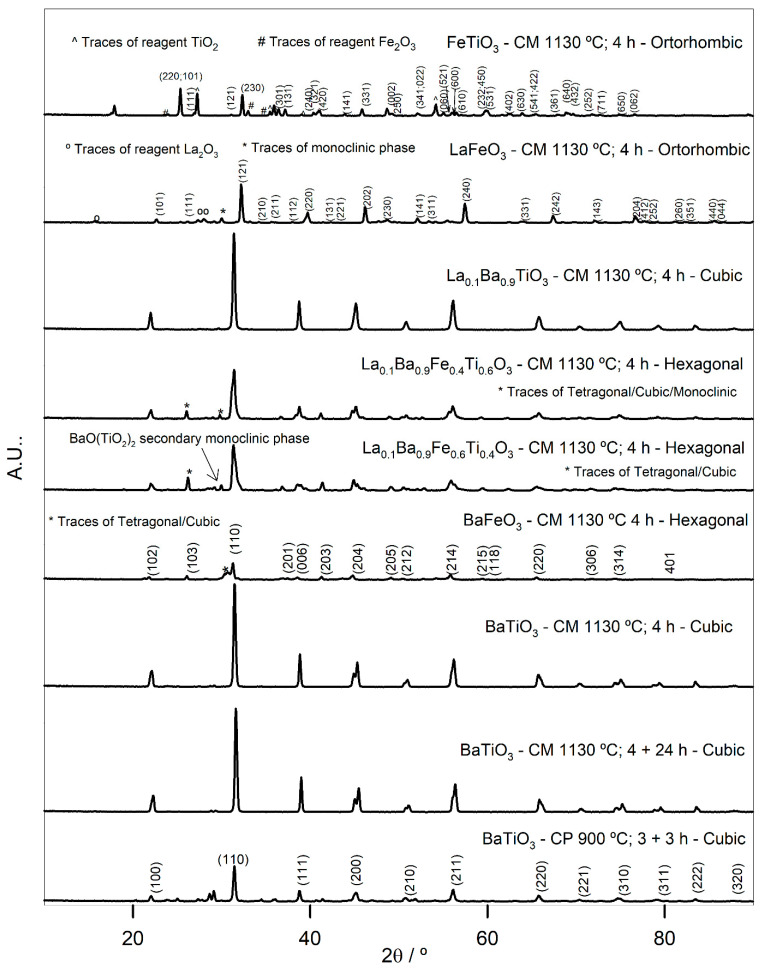
X-Ray diffraction patterns obtained for the different perovskites prepared by different methods.

Figure 1 presents the XRD patterns for perovskite and FeTiO_3_ oxides. BaTiO_3_, prepared either by complex polymerization or ceramic methods, presents a cubic structure with a cell parameter of approximately 0.4 nm (Table 1). The preparation method does not seem to have any influence on the unit cell. Regarding BaTiO_3_ crystallite size, it increases with the annealing temperature, and it is higher for CP, probably because the rearrangement of the ions in the perovskite structure is facilitated by the increase in temperature and also because the use of the CP method involves a much more fluid medium for the powder’s preparation. The molar ratio Ba/Ti, determined by EDS, is smaller when the powder is prepared by CM, moving away from 1. Although EDS presents only a semi-quantitative analysis, this difference may not be disregarded, and it may be related with possible different oxidation states of the transition metal ions in the perovskite structure.

The XRD pattern of the BaFeO_3_ powders prepared by CM shows predominantly characteristic peaks of a hexagonal phase, although there are also other phases present, in smaller quantities, namely tetragonal and cubic. These alternative phases are probably the result of temporary rearrangements in the structure during the solid-state reaction. The Ba/Fe ratio is very close to the theoretical one.

Regarding LaFeO_3_, the XRD pattern shows an orthorhombic structure, with traces of a monoclinic phase and traces of the reagent La_2_O_3_. The presence of the reagent is a possible explanation for the high La/Fe ratio since the reagent may be detected instead of the perovskite. FeTiO_3_, which is not a perovskite, crystallizes in an orthorhombic structure, although it presents traces of the precursors that may explain the low Fe/Ti ratio.

The XRD patterns of the series La_0.1_Ba_0.9_Fe_y_Ti_1−y_O_3_, with y = 0, 0.4, and 0.6, are also presented in Figure 1. For y = 0, the structure is cubic, as for BaTiO_3_, with a similar cell parameter and without secondary phases. This means that the introduction of La in the BaTiO_3_ structure does not promote any distortion. The ratios Ba/La and Ti/La are higher than the theoretical ones. For y = 0.4 and 0.6, the crystalline structure becomes hexagonal, with very similar cell parameters and with traces of other phases. In the case of y = 0.6, there is also the existence of secondary phase BaO(TiO_2_)_2_ (PDF#85-0476) that may explain the smaller ratios of Ba/La, Ti/La, and Ti/Fe for this sample. The introduction of Fe in the La_0.1_Ba_0.9_TiO_3_ structure drastically reduces crystallite size, which becomes like that of BaFeO_3_. This happens because the cubic ideal perovskite structure presents higher crystallites size; the substitution of Ti cations by Fe cations, with a lower ionic ratio, promotes distortion in the ideal perovskite structure that crystallizes in the hexagonal system with a lower crystallite size.

All perovskites present band gap energies between 2.2 and 3.29 eV, indicating that they are suitable as photocatalysts, half of them with visible light. DRX and SEM/EDS were also performed with the perovskite powders after being utilized in the photocatalytic assays, and no significant changes were detected, besides the presence of carbon in the EDS analysis, probably due to the presence of organic matter adsorbed to the powders.

### 3.2. Degradation Assays

The ability of the different prepared perovskites as photocatalysts activated by sunlight was tested with AO7 solutions, utilizing [AO7]_0_ = 5 mg L^−1^ and [catalyst] = 0.5 g L^−1^. Table 2 presents the results obtained in these 50 mL photocatalytic assays. A photolysis result was also introduced to compare. Only BaFeO_3_ and BaTiO_3_-CP present removals in AO7 higher than those observed during the photolysis test. The partial substitution of Ba by La in BaTiO_3_ perovskite apparently leads to the absorption of photons by the powders without activating their catalytic properties, promoting only a reduction in the energy available for photolysis. However, in this last perovskite, when Ti is partially substituted by Fe, as in La_0.1_Ba_0.9_Fe_0.4_Ti_0.6_O_3_ and La_0.1_Ba_0.9_Fe_0.6_Ti_0.4_O_3_, there is the formation of carboxylic acids, showing that AO7 is degraded to SA and AN, which are then oxidized to carboxylic acids, indicating that, after breaking the azo bond, the oxidation reaction proceeds to give smaller, more oxidized, and less toxic products. Another important feature in this series of experiments is that, only for the perovskites La_0.1_Ba_0.9_Fe_0.4_Ti_0.6_O_3_ and La_0.1_Ba_0.9_Fe_0.6_Ti_0.4_O_3_, the formation of oxamic acid is observed, which indicates a different degradation mechanism since, in this case, the amine group does not lead to ammonia formation [37].

Regarding BaTiO_3_-CM and BaTiO_3_-CP, the use of the CP preparation method increases the AO7 removal rate. This increase is probably due to the smaller grain size, with the consequent increase in the surface area of the catalyst. Although the preparation method does not substantially alter the degradation mechanism, as the metabolites obtained are similar, in the case of BaTiO_3_-CP, maleic acid is detected, probably resulting from the subsequent degradation of SA (Table 2).

In the case of photolysis, the presence of AN was not detected by high performance liquid chromatography (HPLC) because, in the presence of sunlight, AN dimerizes, and the dimer was not detected by HPLC. In the presence of the perovskite catalysts, this dimerization should be much smaller since the presence of AN is detected in concentrations much higher than that of SA. A possible explanation for these facts is that only the azo bond is broken by photolysis, followed by the dimerization of the AN. In photocatalysis, dimerization is considerably less, and, after breaking the azo bond, the degradation of SA occurs in a much higher extent than that of AN.

Perovskites BaTiO_3_-CM, BaTiO_3_-CP, and BaFeO_3_-CM had already been tested in the degradation of AO7 but using visible light from a 300 W power lamp instead of natural sunlight [38], and the results were different, particularly for those obtained with the photocatalysts BaTiO_3_-CP and BaFeO_3_-CM. In fact, for the assays run with 500 mg L^−1^ perovskite concentration and 5 mg L^−1^ AO7 initial concentration, the best results with visible light were obtained with BaTiO_3_-CP (80% AO7 removal against the 53% in this work), and with natural sunlight, the best results were attained with BaFeO_3_-CM (74% AO7 removal against the 65% with artificial visible light). This fact shows the difference of using artificial visible light or natural sunlight; this difference is probably ascribed to the UV radiation present in the natural sunlight.

The calcination period in the case of BaTiO_3_-CM seems to have an influence on the AO7 removal rate, and a significant decrease in the AO7 removal rate was observed for the perovskite with a longer calcination time. This is probably due to the increase in the grain size caused by the coalescence of the grains at high temperatures, which can lead to the formation of agglomerates or even sintering, thus reducing the surface area.

Table 3 shows the results of the photolysis and of the adsorption and photocatalysis tests with perovskite BaFeO_3_ using sunlight and different AO7 concentrations. Regarding photolysis, it appears that the increase in the initial AO7 concentration increased the AO7 removal rate in a linear way. In fact, for the 4 h test, if the average photolysis removal rate (v_med_Ph_ = [AO7] removed/4) is plotted as a function of [AO7]_0_, Equation (1) can be obtained, where v_med_Ph_ is in mg L^−1^ h^−1^ and [AO7] in mg L^−1^.

v_med-Ph_ = 0.0812 [AO7]_0_ + 0.172⋯(r^2^ = 0.9999) (1)

Although this calculation is not the most correct, as the correct removal rate should be instantaneous rather than the average of 4 h, it may show that the process can be approximated to first-order kinetics, being the AO7 concentration the rate-determining factor. It is also observed that when AO7 initial concentration increases to 10 mg L^−1^, other metabolites start to form in addition to the usual AN and SA, meaning that AN and SA’s higher formation rate will enhance their further degradation, with the formation of oxamic acid. For the initial concentration of AO7 of 20 mg L^−1^, the AN formation rate should be higher than its dimerization rate, leading to its detection at the end of the assay.

As for the adsorption tests, with a duration of 1 h, there is an increase in the amount adsorbed with the increase in the initial AO7 concentration. If an identical reasoning to that performed for the photolysis data is made, Equation (2) may be obtained, and it represents the average adsorption rate in the period of 1 h as a function of the initial AO7 concentration.

v_med-Ad_ = 0.200 [AO7]_0_ − 0.350⋯(r^2^ = 0.993) (2)

Additionally, for the photocatalysis with BaFeO_3_, an increase in the AO7 removal rate is observed with its initial concentration. If the average rate of photocatalysis for the 4 h test is plotted as a function of the initial AO7 concentration, Equation (3) may be obtained, showing that the average catalytic photodegradation rate is like the average adsorption rate, suggesting that the controlling step of the photocatalytic process may be adsorption, namely the diffusion of the dye molecule to the adsorption site on the catalyst surface.

v_med-PhC_ = 0.227 [AO7]_0_ − 0.202⋯(r^2^ = 0.9999) (3)

Still, for the photocatalysis, it appears that for higher AO7 initial concentrations, there is an evident degradation of the primary metabolites (AN and SA), with the formation of several carboxylic acids, which leads to less toxic final solutions.

Table 4 presents the results of the photocatalysis tests with the BaFeO_3_-CM and BaTiO_3_-CP, with different amounts of perovskite in suspension.

There is an increase in the dye removal rate with BaFeO_3_ concentration in suspension, perhaps because the controlling step is adsorption that increases with the quantity of perovskite in the suspension. The same behavior is no longer true for BaTiO_3_ since there is an increase in the dye removal when the suspension concentration is increased from 250 to 500 mg L^−1^, but a decrease when the concentration is increased from 500 to 750 mg L^−1^. The justification for this behavior may be the existence of a saturation of white powders in this test that will prevent part of the light from being used and being reflected instead. In fact, BaFeO_3_ powders are black. Thus, the increase in photocatalytic activity can be attributed to the increase in active sites, available for the adsorption of dye molecules [38,39]. In the case of BaTiO_3_, the concentration limit that enhances photocatalysis has not been reached. The decrease in activity after increasing the amount of photocatalyst can be attributed to the decrease in the intensity of penetration of light radiation [38,39,40], which can be hampered or even reflected (dispersed), affecting the photosensitive area. Additionally, a reduction in photons can lead to a decrease in the degradation rate [38].

**Figure 2:**
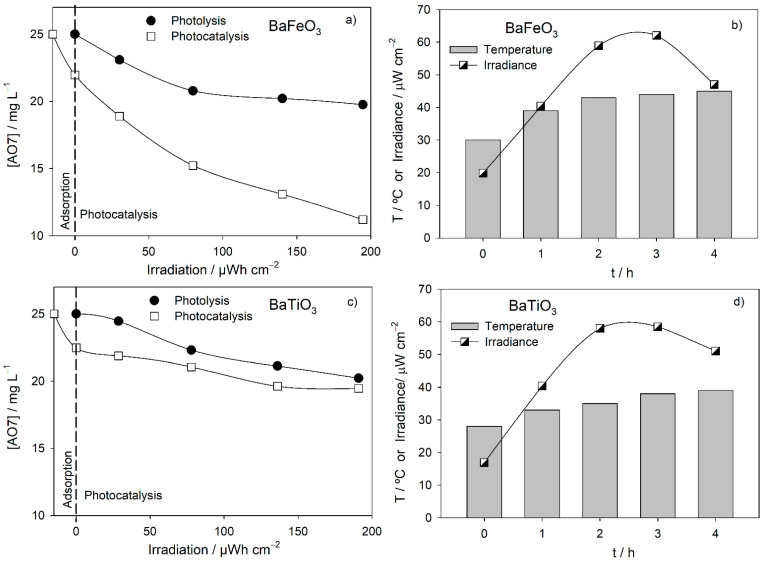
Decay in time of the AO7 concentration, measured as absorbance at 484 nm, for the assays with sunlight using as (**a**) BaFeO_3_ and (**c**) BaTiO_3_ photocatalyst and respective temperature and sunlight irradiance variations (**b**,**d**).

Photocatalysis with BaFeO_3_-CM and BaTiO_3_-CP was repeated with a higher suspension volume (200 mL), and both presented higher AO7 removal rates than those observed during the photolysis tests (Figure 2). In the case of the BaFeO_3_-CM perovskite, the AO7 removal rate during photocatalysis follows a similar trend to that of adsorption, which seems to indicate that adsorption is the controlling step. However, this does not seem to be the case for photocatalysis with BaTiO_3_-CP, with a decrease in the AO7 elimination rate when the catalyst is introduced into the solution after 1 h of adsorption. Regarding the two photolysis tests, AO7 removal is faster in the test shown in Figure 2a,c. A possible explanation for this may be the higher temperature values in the initial phase of the test. As for the decrease in the AO7 removal rate, as observed after 2 h of testing in Figure 2a, it must be due to the marked reduction in irradiance in the final part of the test.

Perovskites can exhibit different photocatalytic behaviors at different temperatures, and BaFeO_3_ has shown better results at higher temperatures. On the other hand, BaTiO_3_ shows a reduction in its photocatalytic activity with increasing temperature [37].

## 4. Conclusions

Several perovskite powders were tested for their catalyst ability in suspension under sunlight for AO7 photodegradation. BaFeO_3_-CM and BaTiO_3_-CP showed the AO7 highest removals rates, originating mainly SA and AN. For the other perovskites tested, AO7 removal rates were lower than that of photolysis. However, with La_0.1_Ba_0.9_Fe_0.4_Ti_0.6_O_3_ and La_0.1_Ba_0.9_Fe_0.6_Ti_0.4_O_3_ as photocatalysts, the breaking of the AO7 azo bond to give SA and AN is followed by their degradation, forming smaller products, such as carboxylic acids, less toxic than the parent compounds, which is an advantage over photolysis.

Regarding the influence of the preparation method, BaTiO_3_-CP shows better photocatalytic activity than BaTiO_3_-CM for AO7 removal. This behavior must be due to the smaller grain size obtained in the complex polymerization method, with the consequent increase in the catalyst’s surface area. For BaTiO_3_-CM, the increase in annealing time led to a reduction in the AO7 removal rate, probably because an increase in the calcination time leads to the coalescence of crystals, forming agglomerates and reducing the surface area available for photocatalysis.

The AO7 degradation with BaFeO_3_ as photocatalyst under sunlight presents a pseudo-first-order reaction rate, being adsorption the controlling step.

## Figures and Tables

**Table 1:** Physical properties of perovskite powders and FeTiO_3_ prepared by different experimental conditions.

Perovskite/Preparation Method ^1^	Crystallite Size/nm	Unit Cell ^2^	Cell Parameters/nm	Eg/eV	EDS	SEM
FeTiO_3_ CM	70.74	Orthorhombic	a = 0.9783 b = 1.0010 c = 0.3745	2.20	Fe/Ti = 0.7	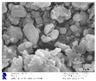
LaFeO_3_ CM	32.15	Orthorhombic	a = 0.5571 b = 0.7842 c = 0.5560	2.30	La/Fe = 1.3	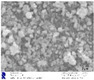
La_0.1_Ba_0.9_TiO_3_ CM	55.12	Cubic	a = 0.4009	3.29	Ba/La = 10.5 Ti/La = 13.5	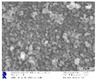
La_0.1_Ba_0.9_Fe_0.4_Ti_0.6_O_3_ CM	36.36	Hexagonal	a = 0.5670 c = 1.4030	3.23	Ba/La = 7.6 Ti/La = 13.5 Ti/Fe = 3.7	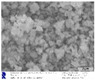
La_0.1_Ba_0.9_Fe_0.6_Ti_0.4_O_3_ CM	38.66	Hexagonal	a = 0.5698 c = 1.4004	2.89	Ba/La = 6.5 Ti/La = 4.9 Ti/Fe = 1.6	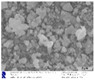
BaFeO_3_ CM	36.37	Hexagonal	a = 0.5673 c = 1.4039	2.96	Ba/Fe = 1.06	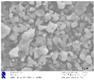
BaTiO_3_ CM	55.16	Cubic	a = 0.4007	3.24	Ba/Ti = 0.85	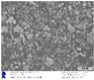
BaTiO_3_ CP	55.76	Cubic	a = 0.4014	3.24	Ba/Ti = 0.96	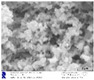

^1^ CM—Ceramic method, annealing time of 4 h at 1130 °C; CP—Complex polymerization method, annealing time of 6 h at 900 °C. ^2^ Holland and Redfern [36] and Program/Software JADE 6 with data base (DRX PDF#cards: 43-1011; 37-1493; 74-1964; 23-1023).

**Table 2:** Results of the photocatalytic assays with sunlight using different perovskites: [catalyst] = 0.5 g L^−1^; [AO7]_0_ = 5 mg L^−1^; Volume = 50 mL; Assay duration—4 h.

Catalyst	AN, SA and Carboxylic Acids Final Concentration ^1^					AO7 Removal/%
	AN	SA	Maleic Acid	Oxamic Acid	Acetic Acid	
-- (Photolysis)	−	++	−	−	−	46
FeTiO_3_, CM_1130 °C, 4 h	++	+	−	−	−	17
LaFeO_3_, CM_1130 °C, 4 h	++	+	−	−	−	36
La_0.1_Ba_0.9_TiO_3_, CM_1130 °C, 4 h	++	+	−	−	−	28
La_0.1_Ba_0.9_Fe_0.4_Ti_0.6_O_3_, CM_1130 °C, 4 h	++	+	++	+++	−	20
La_0.1_Ba_0.9_Fe_0.6_Ti_0.4_O_3_, CM_1130 °C, 4 h	++	+	−	++	−	21
BaFeO_3_, CM_1130 °C, 4 h	++	+	−	−	+	74
BaTiO_3_, CM_1130 °C, 4 h	++	+	−	−	−	28
BaTiO_3_, CM_1130 °C, 4 + 24 h	++	+	−	−	−	24
BaTiO_3_, CP_900 °C, 3 + 3 h	++	+	++	−	−	53

^1^—< 0.001 mg L^−1^; 0.001 mg L^−1^ < + < 0.01 mg L^−1^; 0.01 mg L^−1^ < ++ < 0.1 mg L^−1^; +++ > 0.1 mg L^−1^.

**Table 3:** Results from photolysis, adsorption, and photocatalysis, with BaFeO_3__CM_1130 °C_4 h, utilizing sunlight and different AO7 initial concentration: [catalyst] = 0.5 g L^−1^; Volume = 50 mL; Assay duration—4 h.

Type of Assay	[AO7]_0_/ mg L^−1^	AN, SA and Carboxylic Acids Final Concentration ^1^					AO7 Absolute Removal/mg L^−1^ (AO7 Removal/%)
		AN	SA	Maleic Acid	Oxamic Acid	Acetic Acid	
Photolysis	5	−	−	−	−	−	2.3 (46)
	10	−	++	−	++	−	4.0 (40)
	20	++	++	−	−	−	7.2 (36)
Adsorption	5	n.d. ^2^	n.d.	n.d.	n.d.	n.d.	0.6 (11)
	10	n.d.	n.d.	n.d.	n.d.	n.d.	1.8 (18)
	20	n.d.	n.d.	n.d.	n.d.	n.d.	3.6 (18)
Photocatalysis	5	++	++	−	−	+	3.7 (74)
	10	++	++	++	+++	+	8.4 (84)
	20	++	++	−	+++	+	17.4 (87)

^1^—< 0.001 mg L^−1^; 0.001 mg L^−1^ < + < 0.01 mg ^−1^; 0.01 mg L^−1^ < ++ < 0.1 mg L^−1^; +++ > 0.1 mg L^−1^. ^2^ n.d.—not determined.

**Table 4:** Results from photocatalysis with different perovskites at different concentrations with sunlight: [AO7]_0_ = 5 mg L^−1^; Volume = 50 mL; Assay duration—4 h.

Catalyst	[Catalyst]/ mg L^−1^	AN, SA and Carboxylic Acids Final Concentration ^1^					AO7 Absolute Removal/mg L^−1^ (AO7 Removal/%)
		AN	SA	Maleic Acid	Oxamic Acid	Acetic Acid	
BaFeO_3__CM_1130 °C_4 h	250	++	++	−	++	+	0.3 (6)
	500	++	++	−	−	+	3.7 (74)
	750	++	+	++	++	+	4.6 (92)
BaTiO_3__CP_ 900 °C_3 + 3 h	250	−	+	++	++	−	0.8 (15)
	500	++	+	++	−	−	2.7 (53)
	750	−	+	−	−	−	1.6 (32)

^1^—< 0.001 mg L^−1^; 0.001 mg L^−1^ < + < 0.01 mg L^−1^; 0.01 mg L^−1^ < ++ < 0.1 mg L^−1^; +++ > 0.1 mg L^−1^.

## Data Availability

Data sharing is not applicable to this article.
